# Reliability of Resting-State Microstate Features in Electroencephalography

**DOI:** 10.1371/journal.pone.0114163

**Published:** 2014-12-05

**Authors:** Arjun Khanna, Alvaro Pascual-Leone, Faranak Farzan

**Affiliations:** 1 Berenson-Allen Center for Non-invasive Brain Stimulation, Beth Israel Deaconess Medical Center, Harvard Medical School, Boston, MA, United States of America; 2 Temerty Centre for Therapeutic Brain Intervention, Centre for Addiction and Mental Health, Toronto, Ontario, Canada; University of Bern, Switzerland

## Abstract

**Background:**

Electroencephalographic (EEG) microstate analysis is a method of identifying quasi-stable functional brain states (“microstates”) that are altered in a number of neuropsychiatric disorders, suggesting their potential use as biomarkers of neurophysiological health and disease. However, use of EEG microstates as neurophysiological biomarkers requires assessment of the test-retest reliability of microstate analysis.

**Methods:**

We analyzed resting-state, eyes-closed, 30-channel EEG from 10 healthy subjects over 3 sessions spaced approximately 48 hours apart. We identified four microstate classes and calculated the average duration, frequency, and coverage fraction of these microstates. Using Cronbach's α and the standard error of measurement (SEM) as indicators of reliability, we examined: (1) the test-retest reliability of microstate features using a variety of different approaches; (2) the consistency between TAAHC and *k*-means clustering algorithms; and (3) whether microstate analysis can be reliably conducted with 19 and 8 electrodes.

**Results:**

The approach of identifying a single set of “global” microstate maps showed the highest reliability (mean Cronbach's α>0.8, SEM ≈10% of mean values) compared to microstates derived by each session or each recording. There was notably low reliability in features calculated from maps extracted individually for each recording, suggesting that the analysis is most reliable when maps are held constant. Features were highly consistent across clustering methods (Cronbach's α>0.9). All features had high test-retest reliability with 19 and 8 electrodes.

**Conclusions:**

High test-retest reliability and cross-method consistency of microstate features suggests their potential as biomarkers for assessment of the brain's neurophysiological health.

## Introduction

Neurophysiological impairments may precede the appearance of clinical symptomology in several neuropsychiatric illnesses [1]–[3]. Frequent and longitudinal monitoring of neurophysiological “biomarkers” could enable early detection of disease pathogenesis, and enhance understanding of the neurophysiological impairments underlying these disorders. Thus, there is great interest in developing techniques to detect neurophysiological biomarkers associated with impairments in the brain's functional health.

Electroencephalography (EEG) is a popular and widely used tool that has been explored as one such method capable of detecting the electrophysiology of the brain. EEG detects and records millivolt fluctuations of electric potentials over the cortex with very high temporal resolution [4]. A number of approaches have been proposed to extract features of neurophysiologic relevance from the recording. One such method is to use characteristics of the recorded oscillations to define “states” of the signal that evolve over time. For example, state characteristics such as chaotic complexity [5] or synchronicity [6] have been extracted from resting-state EEG. In this method, brain activity is described in relation to state characteristics, such as the duration or frequency of occurrence of certain states.

Microstate analysis is one such method that defines states of the multichannel EEG signal by spatial topographies of electric potentials over the electrode array. This method was first proposed by Lehmann et al. (1987), who showed that the α frequency band (8–12 Hz) of multichannel resting-state EEG could be parsed into discrete states in this way [7]. When the multichannel resting-state EEG signal is considered as a time series of topographies of electric potentials, two remarkable properties emerge. First, although there are a large number of possible topographies in multichannel recording, a majority of the signal can be represented by surprisingly few maps. Interestingly, most studies of resting-state EEG consistently find the same four archetypal maps that explain more than 70% of the total topographic variance. Second, there is a well-defined temporal structure of these maps, in that a single topography remains dominant for about 80–120 ms before abruptly transitioning to another topography. These periods of quasi-stability of a single topography are “microstates.” Thus, the multichannel EEG signal can be represented by a single time series of microstates alternating among themselves at discrete intervals.

Compared to traditional frequency power EEG analysis, spatial analysis of EEG using microstates has several advantages. Perhaps most importantly, spatial EEG analysis does not assume the EEG signal is a linear dynamical system that can be represented through the Fourier series as a linear function of a set of sine waves. The spatial topography of the EEG signal can be defined at any point in time independently of the preceding or subsequent topography and therefore has millisecond resolution, unlike conventional frequency power analysis that integrates activity over seconds. Indeed, although microstates were initially described in the alpha frequency band, they can in fact be defined within any signal bandwidth; the dominant generator frequency in any given bandwidth dictates the speed of polarity inversions, but resting-state microstate topographies are considered independently of polarity (i.e. two topographies with opposite polarities are considered the same microstate). Microstates are therefore better suited to detect rapid, dynamic activity in large-scale neurocognitive networks than traditional frequency analysis of EEG. These large-scale neural networks, which link spatially distributed cortical areas into functional entities, have been shown to underlie complex neurocognitive activities [8], [9], including those that occur at rest, the so-called “resting-state networks” (RSNs). Accordingly, recent data has indicated that individual microstates may correspond to specific RSNs identified in functional magnetic resonance imaging (fMRI) studies, as there appears to be a temporal correlation between the appearance of microstates and specific RSN activity [10]–[12]. Spatial EEG signal analysis with microstates may be a valuable approach to studying these and other large-scale neurocognitive networks in health and disease.

Consistent with the idea that EEG microstates may reflect the activity of large-scale neurocognitive networks, preliminary reports have found correlations between features of the microstate time series and various cognitive activities, behavioral states, and neuropsychiatric diseases. For example, microstates of certain topographies have a shorter average lifespan in schizophrenia [13], are longer in panic disorder [14], shortened in Alzheimer's disease [15], and appear more frequently in Tourette's syndrome [16]. Neurotropic drugs commonly used to treat neuropsychiatric disease alter microstate features [17], [18]. Microstates vary with cognitive/behavioral states such as drowsiness [19], sleep stages [20], age [21], and even personality characteristics [22]. Pre-stimulus spontaneous EEG microstates also have profound impacts on the electrophysiological [23]–[25] and perceptual [26]–[28] responses to stimuli. These preliminary reports suggest that features of the microstate time series may be related to the neurophysiological basis of these disorders, brain states, and cognitive functions, and can potentially offer insight into the function of the brain in health and disease [29].

The aforementioned cross-sectional studies of microstate features demonstrate intriguing relationships between microstates and disease. To further explore the significance of the microstate time series and its potential utility in the detection of neurophysiological changes underlying disease, longitudinal cohort studies are required to characterize microstate changes over time in individual patients. However, the design of these studies is difficult, because limited information exists about the variance in common microstate features and the test-retest reliability of these values. Furthermore, although there are several different methodological approaches to microstate analysis, few studies have assessed the validity and consistency of these various methods.

In this study, we investigate the test-retest reliability of resting-state EEG microstate features in healthy subjects across three sessions. We extract four of the most common features from the time series, namely, the topography that defined each microstate, the average lifespan of each microstate, the frequency of appearance of each microstate, and the fraction of total time covered by each microstate. Our rationale in choosing these features is based on the fact that these are the most common features examined in previous studies, and changes in each of these features has been associated with one or more neuropsychiatric disorders.

Microstate analysis involves two basic steps: first, a set of microstate topographies is selected, and second, the original data is re-expressed as an alternating sequence of these microstate topographies from which values of interest can be calculated. The first step is usually performed by mathematical clustering of maps in the original data. A single set of microstate topographies can be identified for all subjects (i.e. all of the original data is clustered together), or a unique set of topographies can be generated for subsets of subjects (e.g. different experimental groups may be assigned unique microstates, or each recording may be assigned an individual set of microstates). We tested the consistency of microstate analysis using three different approaches. First, we assumed one set of four *global* maps and identified a single set of four maps that was applied to all subjects across all sessions. Second, we generated a set of topographies *by session* independently. Third, we generated a set of topographies *by recording*, i.e. generated four maps for each individual recording. We also compared two common clustering algorithms (topographic atomize and agglomerate hierarchical clustering and *k*-means clustering) and assessed whether microstate analysis can be performed reliably with as few as 8 electrodes.

## Methods

### Subjects

We studied 10 healthy subjects (mean age: 30±10 yr, 5 females). Subjects were recruited through advertisement in greater Boston area. Exclusion criteria included a self-reported medical illness and history of drug or alcohol abuse. All participants gave their written informed consent and the protocol was approved by the local ethics committee at the Beth Israel Deaconess Medical Centre in accordance with the declaration of Helsinki.

### EEG Recording

Data used in this study was collected as part of a baseline assessment in a larger research study investigating the effect of non-invasive brain stimulation on various cortical processes. Subjects were instructed to sit in a comfortable armchair. Approximately three minutes of resting-state, eyes-closed EEG were recorded in three sessions separated by at least 48 hours. EEG recording was obtained through a 32-channel EEG system (BrainProducts, GmbH) with the CPZ and AFZ electrodes set as reference and ground electrode, respectively. EOG was recorded through two channels placed underneath each eye. The data was sampled at 5 kHz with the online filter setting set to DC to 1 kHz. The skin/electrode impedance was kept below 5 kOhm.

### EEG Preprocessing

Data were imported into MATLAB (The MathWorks. Inc.Natick, MA, USA) for preprocessing. The open source signal processing functions available through the EEGLAB toolbox version 11b [30] were used for data import and preprocessing. The EEG waveforms were epoched into segments of 2 second duration and down sampled to 2 kHz. A notch filter (band-stop: 55–65 Hz) was used to remove the 60 Hz noise. EEG signals were band passed filtered for the frequency range of 1–50 Hz to further minimize contamination by high frequency artifact. The infinite impulse response (IIR) Butterworth filter of second order was employed and both forward and backward filtering was applied (MATLAB function ‘filtfilt’) to maintain a zero phase shift. All epochs were manually reviewed and trials and channels containing eye movements, muscle or any other non-physiological artifact were discarded. The data were then average re-referenced.

### EEG Power Analysis

The EEGLAB function *spectopo* was used to obtain the power spectrum. The absolute and relative power was obtained for delta (1–3.5 Hz), theta (4–7 Hz), alpha (8–12 Hz), and beta (12–30 Hz) frequency bands.

### EEG Microstate Analysis

EEG microstate analysis was conducted using the freely-available CARTOOL software [31]. Preprocessed data were imported into CARTOOL, band passed to 1–30 Hz, and downsampled to 200 Hz before microstate analysis as described below.

**Figure 1:**
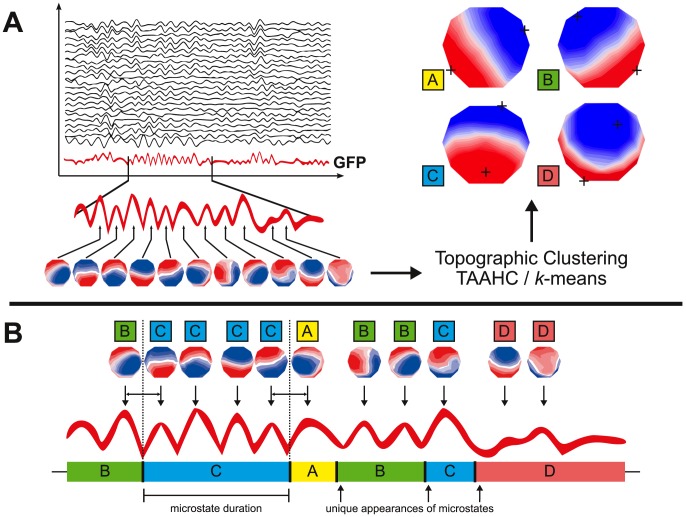
Schematic of the method of microstate analysis and extraction of features of interest from the microstate time series. (**A**) The GFP (drawn in red) is calculated at each instant of the multichannel EEG recording. Peaks of the GFP curve represent moments of highest SNR. At peaks of the GFP curve, the potential recorded at each electrode of the multichannel signal is plotted onto a map of the channel array. This collection of maps is entered into a clustering algorithm (TAAHC or *k*-means clustering), which results in a small number of representative microstate maps that explain a large proportion of the global topographic variance. Four topographies are repeatedly found using this method; these maps are labeled A, B, C, or D in the figure. Crosshairs indicate points of maximum or minimum recorded electric potential. (**B**) The original maps at peaks of the GFP curve are assigned to a microstate class A, B, C, or D based on the degree of correlation with the microstate maps and statistical smoothing of the time series. This reassignment results in a representation of the original multichannel data as an alternating series of microstates A, B, C, and D. A microstate is considered dominant in the time during which all successive original maps are assigned to the same microstate class, starting and ending at the midpoint in time between the last original map of the preceding microstate and the first original map of the following microstate, respectively. Each period of dominance is considered a unique appearance of a microstate. The frequency of a microstate is the number of unique appearances per second. The coverage of each microstate is the fraction of total recording time that each microstate is dominant.

In microstate analysis, the multichannel EEG signal is considered as a series of instantaneous topographies of electric potentials. We identified points with the greatest signal-to-noise ratio (SNR) by calculating the global field power (GFP) of each topography in the time series. The GFP at each point in time is equal to the root mean square across the average-referenced electrodes – equivalently, the standard deviation of the signal at all electrodes:
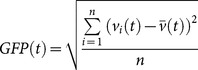
(1)where *v_i_*(*t*) is the voltage at electrode *i* at time *t*, 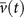 is the mean voltage across all electrodes at time *t*, and *n* is the number of electrodes. Maps that occur at local maxima in the GFP curve – i.e. all points with GFP higher than the preceding and following point – represent instants of highest field strength and greatest SNR. Furthermore, because the field topography remains essentially constant between two local minima of the GFP curve, topographies at GFP maxima are representative of topographies at surrounding points in time [7], [32]. Thus, data reduction of the original signal to points at local GFP maxima is a valid method of enhancing topographic SNR. These maps at local GFP maxima, hereafter referred to as the “original maps,” were extracted and submitted to further analysis (**Figure 1**).

Microstate analysis involves two basic steps – first, a set of microstate maps is identified, and second, this set of maps is fit onto the original data to re-express the multichannel EEG as a sequence of microstates (**Figure 1**).

### 1. Derivation of Maps Globally, by Session, and by Recording

We chose *a priori* to identify four group-level classes in order to remain consistent with the majority of previous studies that also use four microstate classes. We compared three different strategies to identify the four maps that would be used to identify microstates on our original data. In the *global* maps strategy, we clustered original maps from each recording into four maps and then entered this set of 4×30 = 120 maps into another round of clustering to identify four *global* maps that was then fit to all of the original maps. This is similar to the strategy used by Lehmann et al. (2005) [13]. In the *by session* strategy, we clustered original maps from each recording into four maps, and then entered these maps into another round of clustering separately for each session. This resulted in three separate sets of four microstate maps (one for each of three sessions). The maps from each session were fit onto all of the original maps from the respective session. Finally, in the *by recording* strategy, we clustered original maps from each recording into four maps, and used these 30 sets of four maps to fit onto original maps from each respective recording.

#### 1.1. Topographic Atomize and Agglomerate Hierarchical Clustering (TAAHC)

The original maps were submitted into a modified hierarchical clustering algorithm known as the topographic atomize and agglomerate hierarchical clustering (TAAHC) method [33] as implemented by the CARTOOL program [31]. Briefly, all maps submitted to the procedure are initially considered to be independent clusters. In each iteration of the algorithm, the “worst” cluster is identified and split into its constituent maps (“atomized”). The “worst” cluster in each iteration is the one with the lowest summated correlation between each constituent map to the average cluster map. Correlation is analogous to the Pearson product-moment correlation coefficient between two topographies:
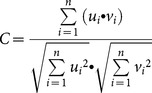
(2)where sums are taken over *i* electrodes. Maps of the “worst” cluster are redistributed (“agglomerated”) to any of the remaining clusters to which they are most strongly correlated. This process is continued until the desired number of clusters is achieved.

**Figure 2:**
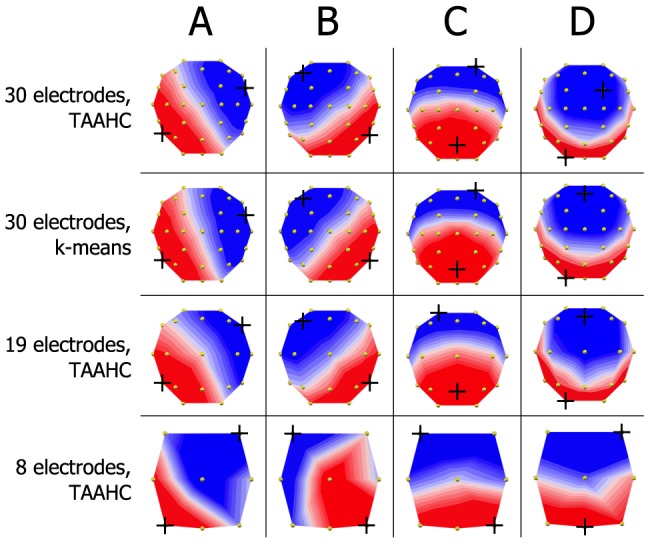
Topographies of microstate A, B, C, and D extracted from various clustering methods and electrode arrays. Crosshairs indicate points of maximum or minimum recorded electric potential. The four microstate classes A, B, C, and D have been reported in a number of prior studies. TAAHC and *k*-means clustering give almost identical microstate maps (first two rows). Because microstates are defined by the topography of electric potentials over the entire scalp, it is possible to identify microstates fewer electrodes. The microstate classes A, B, C, and D are identifiable in 19 and 8 electrode data. These lower-resolution electrode arrays give highly reliable results.

We used TAAHC to cluster the original maps from each subject and session into four cluster maps for each subject and session. In the *by recording* strategy, we fit these four cluster maps onto each recording. In the *by session* strategy, we submitted these 120 cluster maps (4 from each of 10 subjects over 3 sessions) to another round of clustering separately for each session, and fit the maps from each session onto original maps from the respective session. Finally, in the *global* maps strategy, we submitted these 120 cluster maps to the TAAHC algorithm to identify four group-level cluster maps. These four maps were the “microstate maps” and were labeled class A, B, C, and D (**Figure 2**). Cluster maps identified *by recording* and *by session* were labeled A, B, C, and D depending on their degree of correlation with maps A, B, C, and D from the *global* maps strategy. After these labels were assigned to the sets of 4 cluster maps derived *by recording* and *by session*, these unique sets of 4 cluster maps were fit onto the original data.

#### 1.2. Fitting Microstate Maps onto Original Maps

In the final step, original maps are labeled either A, B, C, or D depending on which microstate map has the highest correlation to the original map.

#### 1.3. Extraction of Features from the Microstate Time Series

After labeling at the local maxima of the GFP curve, we could express each of the original signals as an alternating sequence of maps A, B, C, and D. From this microstate time series, we calculated several features. Our outcomes of interest were: (1) topographies of the four cluster maps identified in each clustering strategy, (2) average lifespan of each microstate and all microstates, (3) frequency of appearance of each microstate and all microstates, and (4) fraction of total covered time of each microstate. We calculated these features separately for each subject in each session.

1.3.1. *Average Lifespan of Microstates:* The lifespan of a microstate was calculated as the time during which all successive original maps were assigned to the same microstate class, starting and ending at the midpoint in time between the last original map of the preceding microstate and the first original map of the following microstate, respectively (**Figure 1b**) [13].

1.3.2. *Frequency of Appearance of Microstates:* We calculated the frequency of appearance of each microstate class by counting the number of unique appearances of each microstate divided by the total length of recording (**Figure 1b**) [13].

1.3.3. *Fraction Total Covered Time of Microstates:* We calculated the fraction total covered time (coverage) of each microstate by taking the ratio of the total time spent in each microstate over the total recording time [13]. Note that the coverage of all four microstates can be calculated from their respective average lifespans and frequencies and the total length of recording, i.e. these are not completely independent measures.

### 2. K-Means Clustering

To assess the reliability of microstate analysis performed with the *k*-means clustering algorithm, we repeated the entire analysis, but used *k*-means clustering instead of TAAHC to identify a set of microstate maps. In the *k*-means clustering method, clustering is first initialized and then entered into a convergence loop. During initialization, to find *n* clusters, *n* non-identical maps are randomly selected out of the set of maps entered into the analysis to serve as templates. All maps are then assigned to a cluster seeded by one of the *n* templates based on the degree of correlation to each template. In the convergence loop, all maps in each of the *n* clusters are averaged. These *n* average maps then serve as seeds for new clusters, and all input maps are again assigned to one of *n* clusters based on correlation to cluster seeds. A measure of the quality of current cluster assignment is computed, in our case the global explained variance (GEV):
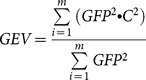
(3)where *m* is the number of original maps. The convergence loop is repeated until the quality of the cluster assignment does not improve. The entire initialization and convergence algorithm is repeated several times to increase the likelihood of finding an optimal set of *n* clusters. The procedure can be repeated for many values of *n*, so the topographies of any number of clusters can be derived. Because the initialization step picks *n* maps to serve as templates randomly, k-means clustering is non-deterministic. To overcome this drawback, the algorithm is repeated 300 times for each value of *n* to minimize run-to-run variance. Maps identified using *k*-means clustering were labeled A, B, C, or D depending on degree of correlation with maps extracting using TAAHC using the *global* maps approach.

### 3. Microstates from Smaller Electrode Arrays

To investigate whether microstate analysis can be reliably conducted using fewer electrodes, we selected 19 electrodes from the original 30-channel recording (AF3, AF4, F7, F3, Fz, F4, F8, T7, C3, C4, T8, Cz, P7, P3, Pz, P4, P8, O1, and O2) and performed TAAHC on these 19-channel data to identify 4 microstates. We also selected 8 electrodes from the original recording (F3, F4, C3, Cz, C4, P3, Pz, and P4) and again performed TAAHC to extract 4 microstates using the *global* maps strategy (**Figure 2**).

### 4. Determining Test-Retest Reliability and Consistency of Microstate Characteristics

All statistical analyses were performed using the SAS software.

Cronbach's α is a well-established measure of the internal consistency and reliability of a test, and was calculated to determine the reliability of microstate characteristics across time, and the consistency of these characteristics across different methods (TAAHC *vs* k-means clustering and various electrode arrays). It is calculated as:
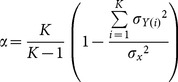
(4)where *K* is the number of repeated tests, 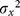 is the variance of the observed data, and 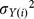 is the variance of component *i* of subject *Y*. Cronbach's α is a measure of reliability relative to between-subject variance. To give a measure of absolute reliability of these features, we also calculated the standard error of measurement (SEM) for microstate characteristics, where appropriate.

#### 4.1. Comparison of Microstate Maps Derived from TAAHC and K-Means Clustering. ing

We tested the four microstates extracted from *k*-means clustering with those extracted from TAAHC for significant differences using topographic analysis of variance (TANOVA). TANOVA is a randomization procedure that uses the GFP of the electrode-by-electrode subtraction between two maps as the test statistic (effect size) of the difference between maps [34]. When the maps being compared are GFP-normalized, the test statistic is equal to the global map dissimilarity (GMD):
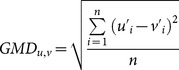
(5)where 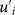 and 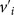 are the potentials at electrode *i* in the GFP-normalized maps being compared.

To derive the distribution of the test statistic under the null hypothesis that there is no difference between maps from *k*-means clustering and TAAHC, we randomly shuffled maps from each subject between two groups and calculated the test statistic between the average group maps, and repeated this procedure 5000 times. The fraction of iterations with a test statistic greater than the one calculated from the actual data was the p value.

#### 4.2. Test-Retest Reliability of Average Microstate Lifespan, Frequency, and Coverage Across 3 Sessions

To determine the test-retest reliability of the average lifespan, frequency, and coverage of each and all microstates across 3 sessions, we calculated Cronbach's α and the SEM of these values from 30-electrode data analyzed with TAAHC clustering, as well as with *k*-means clustering, as described above.

#### 4.3. Consistency of Average Microstate Lifespan, Frequency, and Coverage between TAAHC and K-Means Clustering

To determine the level of consistency between microstate features extracted using TAAHC and *k*-means clustering, we calculated Cronbach's α between the average microstate lifespan, frequency, and coverage measured using microstates extracted by TAAHC and *k*-means clustering.

#### 4.4. Consistency of Average Microstate Duration, Frequency, and Coverage among Channel Arrays with 30, 19, and 8 Electrodes

To determine the level of consistency between microstate features using topographies extracted using channel arrays with 30, 19, and 8 electrodes with TAAHC, we calculated Cronbach's α between the average microstate lifespan, frequency, and coverage measured with these arrays.

## Results

After the data were preprocessed and epochs with artifacts removed, we had a mean of 127.87 seconds of data (SD  = 23.87, range  = 80–204) per recording that were submitted to microstate analysis from which we extracted the “original maps” at local maxima in the GFP curve. We chose *a priori* to cluster the original maps from each session into four microstates. Four microstate maps had a mean GEV of 69.93% (SD  = 3.58, range  = 65.34–77.99) across all recordings using TAAHC.

### 1. Reliability with Maps Derived Globally, by Session, and by Recording

We calculated test-retest reliability of the average lifespan, frequency, and coverage with maps derived *globally*, *by session*, and *by recording* using the TAAHC method. The four *global* maps appear in **Figure 2**. The average lifespan, frequency, and coverage of each microstate calculated using maps derived *globally* appear in **Table 1**, along with Cronbach's α and SEM. The mean Cronbach's α for these values is 0.811. Most values have Cronbach's α>0.7 and SEM is less than 10% of the mean for all values.

**Table 1:** Test-retest reliability of various clustering strategies across 3 sessions separated by at least 48 hours.

Feature/Microstate		TAAHC, 30 electrodes, *Global Maps*			TAAHC, 30 electrodes, *Maps per Session*			TAAHC, 30 electrodes, *Maps per Recording*		
		Mean	α	SEM	Mean	α	SEM	Mean	α	SEM
Average Lifespan (ms)	A	93.75±8.14	0.798	5.51 (5.87%)	98.32±11.48	0.712	8.49 (8.64%)	98.11±11.69	0.739	8.58 (8.74%)
	B	98.96±9.62	0.885	5.23 (5.28%)	94.90±9.36	0.875	5.05 (5.32%)	97.74±9.91	0.723	7.44 (7.61%)
	C	102.35±16.42	0.934	7.11 (6.94%)	101.13±14.26	0.876	8.02 (7.93%)	101.61±9.80	0.835	6.16 (6.07%)
	D	101.59±9.59	0.253	9.15 (9.00%)	102.56±9.57	0.169	9.42 (9.18%)	100.36±11.23	0.653	9.01 (8.98%)
	All	99.95±9.82	0.816	6.41 (6.41%)	100.01±9.74	0.806	6.47 (6.47%)	99.83±9.50	0.834	5.97 (5.98%)
Frequency (appear/sec)	A	2.29±0.37	0.844	0.22 (9.77%)	2.56±0.38	0.584	0.31 (12.23%)	2.47±0.34	0.653	0.27 (11.10%)
	B	2.53±0.32	0.750	0.23 (9.18%)	2.24±0.32	0.708	0.23 (10.28%)	2.51±0.37	0.644	0.30 (11.88%)
	C	2.65±0.36	0.820	0.23 (8.59%)	2.56±0.34	0.220	0.33 (12.96%)	2.65±0.37	0.539	0.32 (12.03%)
	D	2.63±0.45	0.931	0.20 (7.50%)	2.74±0.46	0.928	0.20 (7.48%)	2.48±0.32	0.358	0.30 (11.96%)
	All	10.11±1.08	0.788	0.74 (7.35%)	10.10±1.07	0.779	0.75 (7.41%)	10.11±1.05	0.815	0.68 (6.76%)
Coverage (%)	A	21.28±2.55	0.772	1.76 (8.26%)	25.06±4.23	−0.506	4.14 (16.51%)	24.05±3.35	0.258	3.21 (13.36%)
	B	24.92±3.05	0.873	1.68 (6.75%)	21.14±3.08	0.802	1.73 (8.19%)	24.35±2.96	0.265	2.90 (11.90%)
	C	27.23±6.12	0.976	1.67 (6.12%)	25.86±4.96	0.705	3.82 (14.77%)	26.84±3.74	−0.687	4.15 (15.47%)
	D	26.57±4.13	0.910	2.04 (7.66%)	27.93±4.16	0.901	2.14 (7.67%)	24.76±3.39	−0.748	3.74 (15.10%)

Results of analysis of microstate time series for average lifespan, frequency, and coverage fraction of each microstate. Mean parameter values are presented±standard deviation. α = Cronbach's α, SEM  =  standard error of measurement.

Results from maps derived *by session* and *by recording* are also presented in **Table 1**. For maps derived *by session*, the mean Cronbach's α is 0.648 and SEM is approximately 10% of the mean for all values. For maps derived *by recording*, the mean Cronbach's α is 0.523 and SEM is approximately 10% of the mean.

### 2. Reliability with Maps Derived Using K-Means Clustering

To determine the reliability of the analysis conducted with maps derived using the *k*-means clustering algorithm, we used *k*-means clustering to identify a set of *global* maps and assessed both the reliability of the resulting features over three sessions. The four *global* maps derived using *k*-means appear in **Figure 2**. These four maps had a mean GEV of 70.92% (SD  = 3.65, range  = 65.88–78.70) across all recordings. TANOVA analysis of maps A, B, C, and D extracted using TAAHC and *k*-means reveals no significant difference in the topography of any of the maps (p>0.01). The average lifespan, frequency, and coverage of microstates calculated using these maps appear in **Table 2**. The mean Cronbach's α for these values is 0.830 and SEM is less than 10% of the mean for all values.

**Table 2:** Test-retest reliability of *k*-means clustering analysis and 19- and 8- electrode montages across 3 sessions separated by at least 48 hours.

Feature/Microstate		K-means, 30 electrodes, *Global Maps*			TAAHC, 19 electrodes, *Global Maps*			TAAHC, 8 electrodes, *Global Maps*		
		Mean	α	SEM	Mean	α	SEM	Mean	α	SEM
Average Lifespan (ms)	A	92.36±8.49	0.782	5.90 (6.39%)	92.35±11.17	0.873	6.39 (6.92%)	94.27±9.60	0.864	5.62 (5.97%)
	B	95.03±9.40	0.901	4.79 (5.05%)	96.26±12.77	0.917	6.03 (6.27%)	92.58±10.34	0.918	4.93 (5.32%)
	C	100.53±16.79	0.945	6.71 (6.68%)	105.42±23.59	0.955	8.61 (8.17%)	105.59±15.25	0.924	6.98 (6.61%)
	D	110.36±10.30	0.428	9.27 (8.40%)	102.19±12.29	0.680	9.59 (9.38%)	100.48±17.48	0.957	6.23 (6.20%)
	All	101.13±9.65	0.795	6.52 (6.45%)	100.20±13.96	0.898	7.24 (7.23%)	99.15±12.19	0.940	5.05 (5.10%)
Frequency (appear/sec)	A	2.22±0.37	0.853	0.22 (9.88%)	2.40±0.54	0.792	0.36 (15.06%)	2.58±0.54	0.893	0.28 (10.76%)
	B	2.23±0.29	0.786	0.20 (9.09%)	2.43±0.42	0.879	0.23 (9.58%)	2.44±0.40	0.934	0.17 (7.09%)
	C	2.51±0.37	0.886	0.20 (8.05%)	2.69±0.39	0.801	0.26 (9.75%)	2.70±0.46	0.929	0.20 (7.50%)
	D	3.03±0.49	0.901	0.25 (8.30%)	2.67±0.60	0.936	0.26 (9.63%)	2.52±0.36	0.898	0.19 (7.43%)
	All	9.98±1.04	0.759	0.74 (7.44%)	10.19±1.61	0.840	0.99 (9.75%)	10.24±1.29	0.920	0.61 (5.93%)
Coverage (%)	A	20.27±2.42	0.778	1.67 (8.25%)	21.72±2.67	0.807	1.67 (7.67%)	23.97±3.60	0.740	2.64 (10.99%)
	B	21.03±2.66	0.887	1.42 (6.73%)	23.10±3.88	0.929	1.69 (7.31%)	22.39±3.18	0.892	1.69 (7.57%)
	C	25.47±6.60	0.980	1.63 (6.40%)	28.29±7.27	0.969	2.24 (7.92%)	28.33±5.42	0.923	2.46 (8.70%)
	D	33.23±5.08	0.939	2.13 (6.41%)	26.89±4.89	0.946	1.94 (7.21%)	25.31±6.04	0.973	1.72 (6.78%)

Results of analysis of microstate time series for average lifespan, frequency, and coverage fraction of each microstate. Mean parameter values are presented±standard deviation. α =  Cronbach's α, SEM  =  standard error of measurement.

We also assessed the degree of agreement between values calculated using maps derived from *k*-means clustering and TAAHC in a single session. These Cronbach's α values appear in **Table 2**. All values are above 0.9.

### 3. Reliability with 19 and 8 Electrodes

To determine whether microstate analysis can be reliably conducted with fewer electrodes, we repeated the analysis after selecting 19 and 8 electrodes from the original 30-electrode array using TAAHC and a *global* maps strategy. The four microstate maps derived using 19 and 8 electrodes appear in **Figure 2**. We could clearly identify maps belonging to classes A, B, C, and D in both 19 and 8 electrode data. Average microstate duration, frequency, and coverage from 19 and 8 electrode data appear in **Table 2**. In 19 electrode data, these microstate features have a mean Cronbach's α of 0.873 and SEM approximately 10% of mean values. In 8 electrode data, these features have a mean Cronbach's α of 0.906 and similar SEM.

We also determined the degree of consistency between values extracted from 30, 19, and 8 electrode data in a single session. These Cronbach's α values appear in **Table 3**. Most values are highly consistent across these electrode arrays (average Cronbach's α = 0.834). Notably, the consistency of the average lifespan, frequency, and coverage of microstates C and D tended to be lower than corresponding values for A and B.

**Table 3:** Consistency of microstate features using *global maps* extracted with different clustering algorithms and different electrode arrays.

Feature/Microstate		30 *vs* 19 *vs* 8 electrodes Cronbach's α	TAAHC *vs* K-means Cronbach's α
Average Lifespan (ms)	A	0.953	0.999
	B	0.959	0.998
	C	0.934	0.997
	D	0.833	0.978
	All	0.967	0.998
Frequency (appear/sec)	A	0.943	0.998
	B	0.945	0.984
	C	0.826	0.966
	D	0.794	0.991
	All	0.958	0.998
Coverage (%)	A	0.933	0.998
	B	0.875	0.975
	C	0.751	0.987
	D	−0.230	0.957

### 4. Correlations Among Microstate Features

We calculated the correlation between each pair of microstate features in a single session. As expected, microstate lifespan is inversely correlated with frequency (average correlation R = −0.72 across 4 pairs of microstate lifespans and frequencies) for each individual microstate (e.g. lifespan of microstate A compared to frequency of microstate A, etc.). When comparing these values among all 4 microstates, we found positive correlations among all average lifespans (average correlation R = 0.79) and frequencies (average correlation R = 0.51) (e.g. comparing lifespans of microstates A, B, C, and D) in each individual recording.

### 5. Correlation between Microstate Features and Spectral Power

To explore the relationship between microstate features and the power spectra of EEG recordings, we also calculated the correlation between various microstate features and the absolute and relative power in the delta, theta, alpha, and beta frequency bands averaged across all electrodes. Multiple regression modeling showed that relative beta power is negatively associated (p = 0.0001) and relative alpha power is positively associated (p = 0.0174) with global average microstate duration (R^2^ = 0.92). Conversely, relative alpha power is negatively associated (p = 0.023) and relative beta power is positively associated (p = 0.0003) with overall microstate frequency (R^2^ = 0.89). Power is not significantly associated with coverage fraction of any microstate class. There were no significant correlations between any microstate feature and absolute power in any frequency band.

## Discussion

In this study, we sought to assess the test-retest reliability of resting-state EEG microstate analysis in healthy subjects over time. We used a number of variations of the method to determine the reliability and the degree of consistency among these approaches. This study has four major findings. First, we found that using a *global* set of microstates for all subjects yields average microstate durations, frequencies, and coverage fractions that have high Cronbach's α, indicating excellent test-retest reliability. Second, we found that the use of *global* maps yields results that are in general more reliable than maps identified *by session* or *by recording*. Third, we showed that TAAHC and *k*-means clustering yield highly consistent results. Finally, we showed that microstate analysis can be reliably conducted with as few as 8 electrodes.

The maps A, B, C, and D (**Figure 2**) have been reported by numerous previous studies of resting-state EEG microstate analysis [5], [13], [21], and the average microstate lifespans, frequencies, and coverage fractions calculated in this study are in general agreement with prior studies (**Tables 1** **and** **2**). With few exceptions, most microstate features calculated from a set of *global* maps have Cronbach's α>0.7. Cronbach's α is equivalent to the 3, k intraclass correlation coefficient and is a measure of between-subject variance relative to within-subject variance, i.e. it is a *relative* measure of test-retest reliability. High (generally,>0.7) Cronbach's α value suggests that variance in these values over three spaced sessions is small compared to the distribution of these values in the entire sample. The SEM values reported in **Tables 1** **and** **2** are measures of absolute test-retest reliability, and are approximately 10% of the mean for all values. We conclude that these values have high relative test-retest reliability.

We compared three different strategies for identifying microstate maps to be fit onto each EEG recording. In the *global* maps strategy, we derived a universal set of four maps that was derived using all sessions. We also derived maps *by session*, where maps were re-calculated for each session but held constant for all subjects within each session, and *by recording*, where maps were identified for each individual recording. We found that *global* maps gave the most reliable results. This suggests that microstate analysis is highly sensitive to the topographies that are fit onto the data and used to calculate values of interest. Minor differences in the microstate maps are introduced when maps were recalculated *by session* or *by recording*, which appear to generate within-subject error and lower test-retest reliability. This also indicates that comparison of microstate features, for example between two studies, is most valid when the same maps are used.

Our *a priori* selection of 4 microstates represented our data well, explaining about 70% of the global topographic variance of the data. We chose to cluster our data into 4 microstates to remain consistent with the majority of previous studies. However, methods of deriving a data-driven estimate of the number of microstates required to “best” explain the data have been proposed [33], [35]. The most common of these approaches to minimize the cross-validation (CV) criterion, which is proportional to the ratio between the GEV and the degrees of freedom of the maps [35]. Importantly, the CV criterion is highly sensitive to the number of electrodes used, and some have argued against its use when fewer than 64 electrodes make up the channel array [36]. Another measure, the Krzanowski-Lai criteria, has recently been proposed for microstate analysis [31]. In our data, the CV criterion was on average minimized between 4 and 5 microstates for all recordings; thus, our selection of 4 microstates was similar to the data-driven estimate.

We compared the maps derived using TAAHC and *k*-means clustering to determine the extent of agreement between these two clustering algorithms. We found that TAAHC and *k*-means clustering (iterated 300 times) both give results with excellent test-retest reliability across three sessions. The four *global* maps derived using both methods are highly similar (**Figure 2**). Cronbach's α values for microstate features calculated using maps from these two methods are all above 0.9, indicating that TAAHC and *k*-means give highly consistent results.

Because microstate analysis considers the topography of potentials over the entire cortex in a global representation of brain state, and given the nature of the microstate maps extracted using 30 electrodes, we hypothesized that fewer electrodes could successfully identify the four microstate maps A, B, C, and D and be used to conduct microstate analysis reliably. To test this hypothesis, we eliminated all but 19 and 8 electrodes of our original data and repeated the analysis. We could clearly identify maps representing microstate A, B, C, and D in both 19 and 8 electrode data (**Figure 2**). The results of these analyses were also highly reliable (**Table 2**) and, interestingly, even appeared slightly *more* reliable than 30-electrode data. It is possible that elimination of superfluous electrodes reduced noise in the data and refined the results. We also compared the results from 30, 19, and 8 electrode data in a single session to assess the degree of consistency between these electrode arrays. In general, results from these arrays are consistent (**Table 3**). Notably, results of microstates C and D appear less consistent. This is unsurprising, as the topographies of C and D are similar and are probably more difficult to resolve with fewer electrodes. Nevertheless, these data suggest that microstate analysis can be conduced reliably with as few as 8 electrodes. This may be particularly relevant in the development of microstates as clinically useful biomarkers of disease for longitudinal assessment over time, because it reduces the invasiveness and inconvenience that is otherwise associated with clinical EEG studies.

Within individual recordings, we found positive correlations among the average durations and frequencies of all microstates, suggesting that individuals have a tendency toward relatively longer or shorter and relatively more or less frequent microstates in general. This likely reflects natural inter-subject variance, and may also be a function of age [21]. We were also interested in determining how microstate features relate to power in EEG frequency bands. We found that the global average microstate duration decreases and global average frequency increases with increasing relative power in higher spectral frequencies (beta *vs* alpha bands). These associations are likely due to the fact that increased power in higher frequencies reflects faster cortical oscillations, which gives more frequent local GFP maxima and enables finer resolution of microstate transitions. This also suggests that eyes-open EEG data might be expected to yield shorter and more frequent microstates, as alpha power is reduced in the eyes-open state. Our findings agree somewhat with the findings of Koenig et al. (2002), in which shortening overall average microstate lifespans with age was correlated with increasing relative power in higher frequency bands, although they reported lower correlation values (R^2^<0.44) [21]. We did not find evidence of significant association between the coverage of any microstate and power in any frequency band, in agreement with the findings of Britz el al (2010) [10].

The statistics we present here are an important contribution to the translation of microstates to clinical practice as potential biomarkers of neurophysiological health for longitudinal monitoring in individual patients. Unlike experimental paradigms, in which results from multiple subjects are aggregated to produce estimates of variance that are used to determine the significance of microstate differences between groups (see, for example, [13]–[16], [37]–[40]), assessing the significance of changes in microstates observed across repeated measurements in a single subject requires estimates of measurement reliability. To this end, for example, the SEM can be used to estimate a 95% confidence interval for microstate features outside of which differences in repeated measurements in a single individual can be reasonably attributed to changes in the true value, rather than measurement error [41], [42]. This estimate is given by 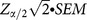, where *Z_α_*_/2_ = 1.96 for a Type I error threshold of 5%. Thus, for the overall microstate duration using 30 electrodes, TAAHC, and a *global* maps strategy, the SEM (6.41 ms from **Table 1**) suggests that, for repeated measurements in a single individual, a change in overall microstate duration of 17.77 ms can be considered significant with 95% confidence. Similarly, reliability can be used to estimate the false-positive and false-negative rate if microstate features are used as decision thresholds [43]. As Cronbach's α is an ICC, it can be used in power calculations in the design of large trials [44]–[46]. We encourage other investigators to use the values reported herein to optimally design future studies.

## Conclusions

In this study, we found that when a *global* set of microstates is used to conduct microstate analysis over multiple sessions, resting-state EEG microstate analysis has high test-retest reliability in healthy subjects as measured by Cronbach's α and SEM. We also determined the consistency of the *k*-means clustering and TAAHC algorithms in extracting microstate maps. Finally, we found that microstate analysis can be reliably conducted with as few as 8 electrodes.

The microstate features we analyzed in this study have been shown to vary in altered cognitive/behavioral states and neuropsychiatric disease, and may be related to the neurophysiological changes that underlie these disorders. The clinical use of microstates as potential biomarkers of disease presupposes within-patient reliability of relevant features, so that changes in the features can reasonably be attributed to changes in neurophysiology. Our aim in this study was to assess the degree of this reliability. Our results indicate good reliability of all the features we examined, and suggest potential value in further exploring microstates as neurophysiological markers of disease in future studies.

## References

[1] Avila MT, McMahon RP, Elliott AR, Thaker GK (2002) Neurophysiological markers of vulnerability to schizophrenia: Sensitivity and specificity of specific quantitative eye movement measures. Journal of Abnormal Psychology 111:259–267. 12003448

[2] Jelic V, Johansson SE, Almkvist O, Shigeta M, Julin P, et al (2000) Quantitative electroencephalography in mild cognitive impairment: longitudinal changes and possible prediction of Alzheimer's disease. Neurobiology of Aging 21:533–540. 10924766 10.1016/s0197-4580(00)00153-6

[3] Ponomareva NV, Fokin VF, Selesneva ND, Voskresenskaia NI (1998) Possible Neurophysiological Markers of Genetic Predisposition to Alzheimer's Disease. Dementia and Geriatric Cognitive Disorders 9:267–273. 9701678 10.1159/000017071

[4] Ingber L, Nunez PL (2011) Neocortical dynamics at multiple scales: EEG standing waves, statistical mechanics, and physical analogs. Mathematical Biosciences 229:160–173. 21167841 10.1016/j.mbs.2010.12.003

[5] Wackermann J, Lehmann D, Dvorak I, Michel CM (1993) Global dimensional complexity of multi-channel EEG indicates change of human brain functional state after a single dose of a nootropic drug. Electroencephalography and Clinical Neurophysiology 86:193–198. 7680995 10.1016/0013-4694(93)90007-i

[6] Carmeli C, Knyazeva MG, Innocenti GM, De Feo O (2005) Assessment of EEG synchronization based on state-space analysis. NeuroImage 25:339–354. 15784413 10.1016/j.neuroimage.2004.11.049

[7] Lehmann D, Ozaki H, Pal I (1987) EEG alpha map series: brain micro-states by space-oriented adaptive segmentation. Electroencephalography and Clinical Neurophysiology 67:271–288. 2441961 10.1016/0013-4694(87)90025-3

[8] Bressler SL (1995) Large-scale cortical networks and cognition. Brain Research Reviews 20:288–304. 7550362 10.1016/0165-0173(94)00016-i

[9] Fuster JM (2006) The cognit: A network model of cortical representation. International Journal of Psychophysiology 60:125–132. 16626831 10.1016/j.ijpsycho.2005.12.015

[10] Britz J, Van De Ville D, Michel CM (2010) BOLD correlates of EEG topography reveal rapid resting-state network dynamics. NeuroImage 52:1162–1170. 20188188 10.1016/j.neuroimage.2010.02.052

[11] Yuan H, Zotev V, Phillips R, Drevets WC, Bodurka J (2012) Spatiotemporal dynamics of the brain at rest — Exploring EEG microstates as electrophysiological signatures of BOLD resting state networks. NeuroImage 60:2062–2072. 22381593 10.1016/j.neuroimage.2012.02.031

[12] Van De Ville D, Britz J, Michel CM (2010) EEG microstate sequences in healthy humans at rest reveal scale-free dynamics. Proceedings of the National Academy of Sciences 107:18179–18184. 10.1073/pnas.1007841107 PMC2964192 20921381

[13] Lehmann D, Faber PL, Galderisi S, Herrmann WM, Kinoshita T, et al (2005) EEG microstate duration and syntax in acute, medication-naïve, first-episode schizophrenia: a multi-center study. Psychiatry Research: Neuroimaging 138:141–156. 15766637 10.1016/j.pscychresns.2004.05.007

[14] Kikuchi M, Koenig T, Munesue T, Hanaoka A, Strik W, et al (2011) EEG Microstate Analysis in Drug-Naive Patients with Panic Disorder. PLoS ONE 6:e22912. 21829554 10.1371/journal.pone.0022912 PMC3146502

[15] Dierks T, Jelic V, Julin P, Maurer K, Wahlund LO, et al (1997) EEG-microstates in mild memory impairment and Alzheimer's disease: possible association with disturbed information processing. Journal of Neural Transmission 104:483–495. 9295180 10.1007/BF01277666

[16] Stevens A, Günther W, Lutzenberger W, Bartels M, Müller N (1996) Abnormal topography of EEG microstates in Gilles de la Tourette syndrome. European Archives of Psychiatry and Clinical Neuroscience 246:310–316. 8908413 10.1007/BF02189024

[17] Lehmann D, Wackermann J, Michel CM, Koenig T (1993) Space-oriented EEG segmentation reveals changes in brain electric field maps under the influence of a nootropic drug. Psychiatry Research: Neuroimaging 50:275–282. 8177925 10.1016/0925-4927(93)90005-3

[18] Yoshimura M, Koenig T, Irisawa S, Isotani T, Yamada K, et al (2007) A pharmaco-EEG study on antipsychotic drugs in healthy volunteers. Psychopharmacology 191:995–1004. 17333135 10.1007/s00213-007-0737-8

[19] Cantero J, Atienza M, Salas R, Gómez C (1999) Brain Spatial Microstates of Human Spontaneous Alpha Activity in Relaxed Wakefulness, Drowsiness Period, and REM Sleep. Brain Topography 11:257–263. 10449257 10.1023/a:1022213302688

[20] Brodbeck V, Kuhn A, von Wegner F, Morzelewski A, Tagliazucchi E, et al (2012) EEG microstates of wakefulness and NREM sleep. Neuroimage 62:2129–2139. 22658975 10.1016/j.neuroimage.2012.05.060

[21] Koenig T, Prichep L, Lehmann D, Sosa PV, Braeker E, et al (2002) Millisecond by Millisecond, Year by Year: Normative EEG Microstates and Developmental Stages. NeuroImage 16:41–48. 11969316 10.1006/nimg.2002.1070

[22] Schlegel F, Lehmann D, Faber P, Milz P, Gianotti LR (2012) EEG Microstates During Resting Represent Personality Differences. Brain Topography 25:20–26. 21644026 10.1007/s10548-011-0189-7

[23] Kondakor I, Lehmann D, Michel CM, Brandeis D, Kochi K, et al (1997) Prestimulus EEG microstates influence visual event-related potential microstates in field maps with 47 channels. J Neural Transm 104:161–173. 9203079 10.1007/BF01273178

[24] Kondakor I, Pascual-Marqui RD, Michel CM, Lehmann D (1995) Event-related potential map differences depend on the prestimulus microstates. J Med Eng Technol 19:66–69. 7494212 10.3109/03091909509030277

[25] Lehmann D, Michel CM, Pal I, Pascual-Marqui RD (1994) Event-related potential maps depend on prestimulus brain electric microstate map. Int J Neurosci 74:239–248. 7928108 10.3109/00207459408987242

[26] Britz J, Landis T, Michel CM (2009) Right Parietal Brain Activity Precedes Perceptual Alternation of Bistable Stimuli. Cerebral Cortex 19:55–65. 18424780 10.1093/cercor/bhn056

[27] Mohr C, Michel CM, Lantz G, Ortigue S, Viaud-Delmon I, et al (2005) Brain state-dependent functional hemispheric specialization in men but not in women. Cereb Cortex 15:1451–1458. 15689523 10.1093/cercor/bhi025

[28] Muller TJ, Koenig T, Wackermann J, Kalus P, Fallgatter A, et al (2005) Subsecond changes of global brain state in illusory multistable motion perception. J Neural Transm 112:565–576. 15340871 10.1007/s00702-004-0194-z

[29] Lehmann D, Strik WK, Henggeler B, Koenig T, Koukkou M (1998) Brain electric microstates and momentary conscious mind states as building blocks of spontaneous thinking: I. Visual imagery and abstract thoughts. International Journal of Psychophysiology 29:1–11. 9641243 10.1016/s0167-8760(97)00098-6

[30] Delorme A, Makeig S (2004) EEGLAB: an open source toolbox for analysis of single-trial EEG dynamics including independent component analysis. J Neurosci Methods 134:9–21. 15102499 10.1016/j.jneumeth.2003.10.009

[31] Brunet D, Murray MM, Michel CM (2011) Spatiotemporal analysis of multichannel EEG: CARTOOL. Intell Neuroscience 2011:1–15. 10.1155/2011/813870 PMC3022183 21253358

[32] Lehmann D, Skrandies W (1980) Reference-free identification of components of checkerboard-evoked multichannel potential fields. Electroencephalography and Clinical Neurophysiology 48:609–621. 6155251 10.1016/0013-4694(80)90419-8

[33] Tibshirani R, Walther G (2005) Cluster Validation by Prediction Strength. Journal of Computational and Graphical Statistics 14:511–528.

[34] Koenig T, Melie-Garcia L (2009) Statistical analysis of multichannel scalp field data. In: Koenig T, Melie-Garcia L, editors. Electrical Neuroimaging. Cambridge, United Kingdom: Cambridge University Press. pp. 169–190.

[35] Pascual-Marqui RD, Michel CM, Lehmann D (1995) Segmentation of brain electrical activity into microstates: model estimation and validation. Biomedical Engineering, IEEE Transactions on 42:658–665. 10.1109/10.391164 7622149

[36] Murray M, Brunet D, Michel C (2008) Topographic ERP Analyses: A Step-by-Step Tutorial Review. Brain Topography 20:249–264. 18347966 10.1007/s10548-008-0054-5

[37] Irisawa S, Isotani T, Yagyu T, Morita S, Nishida K, et al (2006) Increased Omega Complexity and Decreased Microstate Duration in Nonmedicated Schizophrenic Patients. Neuropsychobiology 54:134–139. 17199099 10.1159/000098264

[38] Nishida K, Morishima Y, Yoshimura M, Isotani T, Irisawa S, et al (2013) EEG microstates associated with salience and frontoparietal networks in frontotemporal dementia, schizophrenia and Alzheimer's disease. Clinical Neurophysiology 124:1106–1114. 23403263 10.1016/j.clinph.2013.01.005

[39] Strik WK, Chiaramonti R, Muscas GC, Paganini M, Mueller TJ, et al (1997) Decreased EEG microstate duration and anteriorisation of the brain electrical fields in mild and moderate dementia of the Alzheimer type. Psychiatry Research: Neuroimaging 75:183–191. 9437775 10.1016/s0925-4927(97)00054-1

[40] Strik WK, Dierks T, Becker T, Lehmann D (1995) Larger topographical variance and decreased duration of brain electric microstates in depression. Journal of Neural Transmission/General Section JNT 99:213–222. 10.1007/BF01271480 8579806

[41] Roebroeck ME, Harlaar J, Lankhorst GJ (1993) The Application of Generalizability Theory to Reliability Assessment: An Illustration Using Isometric Force Measurements. Physical Therapy 73:386–395. 8497513 10.1093/ptj/73.6.386

[42] Eliasziw M, Young SL, Woodbury MG, Fryday-Field K (1994) Statistical Methodology for the Concurrent Assessment of Interrater and Intrarater Reliability: Using Goniometric Measurements as an Example. Physical Therapy 74:777–788. 8047565 10.1093/ptj/74.8.777

[43] Charter RA, Feldt LS (2001) Meaning of Reliability in Terms of Correct and Incorrect Clinical Decisions: The Art of Decision Making is Still Alive. Journal of Clinical and Experimental Neuropsychology 23:530–537. 11780951 10.1076/jcen.23.4.530.1227

[44] Fleiss JL (2011) Design and Analysis of Clinical Experiments: Wiley.

[45] Shrout PE (1998) Measurement reliability and agreement in psychiatry. Statistical Methods in Medical Research 7:301–317. 9803527 10.1177/096228029800700306

[46] Perkins DO, Wyatt RJ, Bartko JJ (2000) Penny-wise and pound-foolish: the impact of measurement error on sample size requirements in clinical trials. Biological Psychiatry 47:762–766. 10773186 10.1016/s0006-3223(00)00837-4

